# A Self-Controlled Benchmark of Retrieval-Augmented Generation for Large Language Models on Clinical Guideline Questions

**DOI:** 10.3390/diagnostics16152456

**Published:** 2026-08-04

**Authors:** Andreas Vollmer, Lara Schorn, Felix Schrader, Norbert Kübler, Christoph Sproll, Michael Vollmer, Daman Deep Singh, Babak Saravi

**Affiliations:** 1 Department of Oral and Maxillofacial Plastic Surgery, University Hospital of Wuerzburg, 97070 Wuerzburg, Germany; vollmer_a@ukw.de; 2 Department of Oral, Maxillofacial and Facial Plastic Surgery, Medical Faculty and University Hospital Duesseldorf, Heinrich-Heine-University Duesseldorf, 40225 Duesseldorf, Germany; lara.schorn@med.uni-duesseldorf.de (L.S.); felix.schrader@med.uni-duesseldorf.de (F.S.); kuebler@med.uni-duesseldorf.de (N.K.); christoph.sproll@med.uni-duesseldorf.de (C.S.); damandeep.singh@med.uni-duesseldorf.de (D.D.S.); 3 Department of Oral and Maxillofacial Surgery, Tuebingen University Hospital, Osianderstrasse 2-8, 72076 Tuebingen, Germany; michael.vollmer@med.uni-tuebingen.de

**Keywords:** large language models, retrieval-augmented generation, clinical decision support, oral cavity carcinoma, clinical practice guidelines, artificial intelligence, hallucination, benchmark study, oncology, evidence-based medicine

## Abstract

**Background/Objectives**: Large language models (LLMs) show promise for clinical decision support, yet their accuracy in interpreting specialized medical guidelines remains uncertain. Retrieval-augmented generation (RAG) may enhance performance by grounding responses in authoritative knowledge bases. This study aimed to compare the accuracy, comprehensiveness, and safety of RAG-enhanced versus standard LLMs for answering clinical questions derived from the German S3 guideline for oral cavity carcinoma. **Methods**: We conducted a prospective, single-blind benchmark study evaluating six LLMs: one RAG-enhanced model (Custom GPT with guideline access), one consensus-based model (ConsensusGPT), and four standard models (DeepSeek-V3.2, Mistral Small 3.2, Qwen3-Next-80B, GPT-OSS-120B). Fifty clinical questions covering 17 guideline domains were presented to each model three times, yielding 900 evaluations. Three expert reviewers assessed responses using 5-point Likert scales for accuracy, comprehensiveness, and clarity, under a single-blind procedure, the effectiveness of which was tested by a pre-specified manipulation check. We then ran a paired within-model experiment in which each base model was queried with and without guideline access through a transparent, openly released retrieval pipeline, and scored every response with a condition-blind automated judge alongside deterministic retrieval metrics computed from the logs. Secondary outcomes included hallucination rates and guideline citation behavior. Inter-rater reliability was assessed using intraclass correlation coefficients (ICCs). **Results**: In a paired within-model design that held each base model fixed, adding transparent guideline retrieval improved accuracy—significantly in the three weaker open-weight models (Mistral, Qwen3, and GPT-OSS) and directionally in the already-strong DeepSeek and GPT-5 bases. Because a pre-specified blinding check found that experts could still identify retrieval-augmented answers with 98.5% accuracy, we anchored causal interpretation on measures that do not depend on the human raters, ranked by their independence: deterministic, log-derived retrieval metrics first, and then an automated, condition-blind LLM judge, whose agreement with the experts (Spearman ρ = 0.81, 95.7% within-one agreement) establishes shared calibration rather than independence from their bias. Deterministically from the retrieval logs, citation groundedness rose from 0% to 51–89% and retrieval recall@5 was 92%. On the judge, content-level hallucination fell from 42% to 4% and accuracy rose by a pooled +0.64 points (95% CI 0.47–0.80); the accuracy gain persisted after adjustment for response length (+0.48, 95% CI 0.22–0.73), which retrieval shortened rather than lengthened. The accuracy gain was large for weaker base models and small or non-significant for already-strong ones, whereas the hallucination and auditability gains were consistent across all models. The human ratings reproduced the judge’s accuracy effect (+0.61, 95% CI 0.49–0.74), and GPT-5 run through the transparent pipeline showed no significant difference from the proprietary Custom GPT (judge accuracy 4.48 vs. 4.58). **Conclusions**: Guideline retrieval yields a reproducible, largely base-independent improvement in the safety and auditability of LLM answers to clinical guideline questions, with accuracy gains concentrated in weaker base models. Because retrieval-augmented answers are recognizable to experts, rigorous evaluation should rely on rater-independent measures, and residual hallucination continues to require human oversight.

## 1. Introduction

Large language models (LLMs) have emerged as transformative tools in healthcare, demonstrating unprecedented capabilities in natural language understanding and generation that hold significant promise for clinical decision support [1,2]. Recent systematic reviews have documented a dramatic surge in LLM research within healthcare, with publications increasing from a single study in 2019 to over 550 in 2024, reflecting the expanding recognition of their clinical potential [3]. These models have shown promising applications across diagnostic assistance, treatment recommendations, medical education, and patient communication, with some studies reporting that LLMs can outperform junior trainees in specific medical knowledge assessments [4,5]. However, the accuracy and reliability of LLM-generated medical advice remain subjects of ongoing investigation and concern, particularly regarding their adherence to evidence-based clinical guidelines [6].

Clinical practice guidelines represent the gold standard for evidence-based medical decision-making, synthesizing the best available evidence with expert consensus to provide actionable recommendations. Oral cavity carcinoma presents a significant global health burden, ranking as the 16th most common cancer worldwide with approximately 390,000 new cases annually and an incidence expected to increase by 30% by 2030 [7,8]. The five-year survival rate remains approximately 40–50%, highlighting the critical importance of guideline-concordant care in improving patient outcomes [9]. The German S3 guidelines, representing the highest methodological quality level in the national classification system, provide comprehensive recommendations covering the complete patient journey from risk factor identification through diagnosis, multimodal treatment, and long-term follow-up [10].

A critical limitation of standard LLMs is their reliance on knowledge acquired during pre-training, which may be outdated, incomplete, or incorrect for specialized medical domains [11]. Retrieval-augmented generation (RAG) has emerged as a promising approach to address this limitation by enabling models to access and cite authoritative knowledge bases at inference time [12,13]. Recent meta-analyses have demonstrated that RAG significantly improves LLM performance in biomedical applications, with a pooled effect size of 1.35 (95% CI 1.19–1.53) compared to non-augmented models [14]. Studies evaluating RAG for guideline interpretation have shown that augmented models can achieve accuracy rates exceeding 96%, significantly outperforming both standard LLMs and human-generated responses in specific clinical scenarios [15]. However, comparative data on RAG versus non-RAG LLM performance for medical guideline adherence across comprehensive question sets remain limited.

Furthermore, the phenomenon of “hallucination”—where LLMs generate plausible-sounding but factually incorrect or fabricated information—poses significant risks in medical contexts [16]. Previous studies have documented substantial variability in hallucination rates, ranging from 1.5% in optimized clinical documentation workflows to over 28% for citation generation tasks, with some models producing fabricated references in up to 91% of systematic review queries [17,18,19]. In adversarial testing scenarios, LLMs have exhibited hallucination rates of 50–82%, generating false laboratory values or describing non-existent medical conditions [20]. While prompt engineering and RAG can reduce these errors, they do not eliminate them entirely, underscoring the continued necessity for human oversight in clinical AI applications [21].

We therefore conducted a prospective benchmark study to evaluate the performance of six LLMs in answering clinical questions derived from the German S3 guideline for oral cavity carcinoma. Our primary objectives were to (1) compare accuracy, comprehensiveness, and clarity across models with and without retrieval augmentation; (2) quantify hallucination rates in guideline-based medical question answering; (3) evaluate guideline citation behavior as a measure of response transparency and verifiability; and (4) assess performance variation across question difficulty levels and clinical domains. To our knowledge, this represents one of the first self-controlled benchmarks to isolate the effect of guideline retrieval within each base model against a comprehensive S3-level clinical guideline, evaluated with an automated judge alongside expert human raters.

## 2. Materials and Methods

### 2.1. Study Design and Setting

We conducted a prospective, single-blind, comparative benchmark study to evaluate the performance of six large language models (LLMs) in answering clinical questions derived from the German S3 guideline for oral cavity carcinoma. The primary objective was to compare the accuracy, comprehensiveness, and clarity of LLM-generated responses against gold-standard answers derived from the official S3 guideline. Secondary objectives included evaluation of hallucination rates, guideline citation behavior, and performance variation across question difficulty levels and clinical domains.

### 2.2. Reference Standard: S3 Guideline

The reference standard was the German S3-Leitlinie “Diagnostik und Therapie des Mundhöhlenkarzinoms” (AWMF Register Number 007-100OL, Version March 2021). S3 guidelines represent the highest methodological quality level in the German guideline classification system, featuring systematic evidence searches, formal consensus processes, and explicit recommendation grades. The guideline was developed by 24 medical societies and organizations under the coordination of the German Society for Oral and Maxillofacial Surgery (DGMKG) and the German Cancer Society (DKG).

Recommendation grades in the guideline follow the GRADE system: Grade A (“shall”/strong recommendation, based on high-quality evidence), Grade B (“should”/moderate recommendation), Grade 0 (“may”/open recommendation), and Expert Consensus (EK, based on clinical expertise when evidence is lacking). Evidence levels range from 1++ (high-quality meta-analyses) to 4 (expert opinion).

### 2.3. Question Development and Validation

#### 2.3.1. Question Generation

A total of 50 clinical questions were systematically developed to cover the full scope of the S3 guideline. Questions were generated through iterative expert consultation involving two oral and maxillofacial surgeons, one oncologist, and one medical informatician. The question set was designed to represent all major clinical decision points encountered in the management of oral cavity carcinoma, from initial diagnosis through follow-up care.

Questions were categorized into four types: (1) factual questions testing recall of specific guideline content (n = 22, 44%), (2) recommendation-level questions requiring identification of evidence grades and recommendation strengths (n = 17, 34%), (3) clinical decision questions requiring synthesis of multiple guideline aspects (n = 6, 12%), and (4) case vignettes presenting realistic clinical scenarios (n = 5, 10%) (Table 1).

#### 2.3.2. Guideline Domain Coverage

Questions were mapped to 17 distinct guideline domains to ensure comprehensive coverage: (1) Risk factors, (2) Screening and prevention, (3) Primary diagnostics, (4) Imaging procedures, (5) Biopsy and histopathology, (6) TNM staging, (7) General therapy principles, (8) Surgical therapy, (9) Neck dissection, (10) Reconstruction, (11) Radiotherapy, (12) Radiotherapy side effects, (13) Systemic therapy, (14) Recurrence management, (15) Palliative care, (16) Follow-up, and (17) Rehabilitation. Each domain was represented by 2–4 questions to ensure adequate sampling.

#### 2.3.3. Gold Standard Answer Development

For each question, a gold standard answer was extracted verbatim or paraphrased from the S3 guideline by two independent reviewers. Discrepancies were resolved through discussion with a senior consultant. Gold standard answers included specific guideline references (chapter, recommendation number), evidence levels, and recommendation grades where applicable. All gold standards were verified against the original guideline PDF prior to data collection.

### 2.4. Large Language Model Selection

Six LLMs were selected based on the following criteria: (1) public availability for research use, (2) demonstrated performance on medical benchmarks, (3) representation of different architectural approaches, and (4) inclusion of at least one retrieval-augmented generation (RAG) system. The final selection comprised six systems for the original comparison (Table 2); a seventh, GPT-5-chat, was added as the base for the paired within-model experiment (Section 3.3.4).

The models were chosen to represent three deployment paradigms relevant to the clinical setting rather than to sample the model landscape exhaustively: a guideline-grounded retrieval-augmented system (Custom GPT), a literature-augmented system (ConsensusGPT), and unaugmented base models. The two augmented systems share an identical GPT-5 base, enabling a same-base comparison, while the four open-weight checkpoints span four independent developers and a 24B–685B parameter range through a single, version-pinned API. Additional widely used systems (e.g., Claude, Gemini, GPT-4o, Llama-3.x) were not included, primarily to manage cost and keep the study scope tractable; the selection is therefore paradigm-representative rather than exhaustive, and broader model coverage is a direction for future work.

Model versions and access: Custom GPT and ConsensusGPT were built on GPT-5 via the OpenAI interface; the four open-weight checkpoints were accessed via the DeepInfra API (deepseek-ai/DeepSeek-V3.2-Exp, mistralai/Mistral-Small-3.2-24B-Instruct-2506, Qwen/Qwen3-Next-80B-A3B-Instruct, openai/gpt-oss-120b); the transparent-pipeline arm used gpt-5-chat-latest (OpenAI). Open-weight and hosted models were queried October–November 2025, and the transparent-pipeline and paired arms in July 2026.

#### 2.4.1. Model Configuration and Prompting

All models were queried using identical system prompts and inference parameters to ensure comparability. The temperature parameter was set to 0.2 (low) to minimize response variability while maintaining some natural language variation where possible. Maximum token length was set to 2000 tokens to accommodate comprehensive responses. For API-accessed models (DeepSeek, GPT-OSS, Qwen3, Mistral), queries were executed via the DeepInfra inference platform using the OpenAI-compatible API endpoint.

The standardized system prompt was (English translation):

*“You are a medical expert in oral and maxillofacial surgery with comprehensive knowledge of the diagnosis and treatment of oral cavity carcinoma. Answer the following question precisely and evidence-based. Refer to current medical guidelines and scientific evidence. Provide specific recommendations with recommendation grades where possible. Name relevant risk factors, diagnostic procedures, or therapy options. If you are uncertain, communicate this transparently. Adhere to evidence-based medicine.”*

#### 2.4.2. Custom GPT Configuration

The Custom GPT was configured using OpenAI’s GPT Builder interface with the complete S3 guideline PDF (007-100OL, 156 pages) uploaded as a knowledge base. The RAG system was instructed to retrieve relevant guideline sections before generating responses and to cite specific chapter and recommendation numbers. Web browsing, DALL-E image generation, and code interpreter capabilities were disabled to ensure responses were derived solely from the uploaded guideline. Response length was calibrated to match other models (100–400 words).

#### 2.4.3. Transparent Open-Weight RAG Pipeline

To isolate the effect of retrieval from base-model capability and to provide a reproducible alternative to the proprietary GPT-Builder configuration, we implemented an open retrieval-augmented generation pipeline over the identical S3 guideline PDF and applied it to each comparator in a paired design (base vs. base + guideline retrieval). The guideline was parsed with PyMuPDF and segmented into structure-aware chunks of approximately 400 tokens with 64-token overlap. Chunks were embedded with the open multilingual model BAAI/bge-m3 and indexed in FAISS using exact cosine search; a parallel BM25 index provided lexical retrieval. For each query, dense and lexical candidates were combined by reciprocal-rank fusion (k = 60) and the top 20 reranked with the cross-encoder BAAI/bge-reranker-v2-m3; the five highest-scoring passages were injected under the identical system prompt. Generation used temperature 0.2, a 2000-token limit, and three replicates, and every retrieved passage was logged. The same pipeline was applied to GPT-5 (base with tools disabled, and with transparent retrieval). Full reproducibility applies to the retrieval stack and to the open-weight models on pinned weights; hosted generation is bounded by API and model-version stability, and exact model snapshot identifiers and access dates are reported in Table 2. Because the GPT-Builder retrieval mechanism is proprietary, any internal values correspond to OpenAI’s documented file-search defaults, which may not apply to GPT-Builder and cannot be independently verified. The complete pipeline, index, chunked text, prompts, and per-query retrieval logs are publicly released (https://doi.org/10.5281/zenodo.21342659).

### 2.5. Data Collection Procedures

#### 2.5.1. Query Execution

Data collection was performed between 11 October 2025 and 28 November 2025. Each of the 50 questions was presented to each of the six LLMs three times (replicates) to assess response consistency, yielding a total of 900 query-response pairs (50 × 6 × 3). Queries were executed in randomized order within each model to minimize potential order effects. A minimum interval of 1.5 s was enforced between consecutive API calls to respect rate limits and ensure independent responses.

For API-accessed models, a Python 3.11 script implementing exponential backoff retry logic (maximum 3 retries) handled transient failures. Responses were automatically saved to a structured dataset after each query, with timestamps and token usage metadata. For Custom GPT and ConsensusGPT, queries were executed manually through the respective web interfaces, with responses copied verbatim to the dataset.

#### 2.5.2. Blinding Procedures

A single-blind design was implemented to minimize evaluation bias. Reviewers assessed LLM responses without knowledge of which model generated each response. The dataset presented to reviewers contained only question text, LLM response, and gold standard answer, with model identifiers replaced by randomly assigned codes (A–F). The randomization key was stored separately and revealed only after all evaluations were complete. Reviewers were instructed not to attempt to identify models based on response characteristics.

### 2.6. Evaluation Framework

#### 2.6.1. Reviewer Selection and Training

Three independent reviewers with clinical expertise in oral and maxillofacial surgery evaluated all LLM responses. Reviewers were selected based on: (1) board certification or advanced training in oral and maxillofacial surgery, (2) familiarity with the S3 guideline, and (3) no involvement in question development or LLM configuration. Prior to the main evaluation, reviewers completed a calibration session involving joint assessment of 10 pilot responses with discussion of rating criteria and resolution of discrepancies.

#### 2.6.2. Assessment Dimensions

Each response was evaluated on three primary dimensions using a 5-point Likert scale (1 = lowest, 5 = highest) (Table 3).

#### 2.6.3. Secondary Outcome Measures

In addition to the primary Likert-scale assessments, reviewers evaluated two binary outcomes for each response:

Hallucination Detection: Defined as the presence of fabricated information, invented citations, or factual claims contradicting the gold standard or established medical knowledge. Responses were marked “Yes” if any hallucinated content was identified, regardless of the proportion of accurate content.

Source Citation: Defined as explicit reference to the S3 guideline, AWMF, specific chapter numbers, recommendation identifiers (e.g., “Recommendation 4.3”), or evidence levels (e.g., “LoE 2+”). Generic statements about “guidelines” without specific attribution did not qualify.

#### 2.6.4. Blinding Normalization and Manipulation Check

The single-blind design was vulnerable to a citation-formatting confound (the RAG model cited sources in 89.3% of responses vs. 6.0–25.3% for other models). We applied a deterministic normalizer that deleted all source-identifying citations and provided-context and version references uniformly across arms; a token-level audit and a manual spot-check of 50 responses confirmed that only citation and meta tokens were removed and no clinical content was altered. All 650 responses of the paired round—including Custom GPT and ConsensusGPT—were re-randomized and re-scored on the normalized text by the three blinded experts. A pre-specified manipulation check then asked raters to guess, for each response, whether the answer used guideline access. Across the 500 base and RAG responses (1498 guesses), raters identified the condition with 98.5% accuracy (chance 50%); blinding was therefore not achieved in this study. This reflects both residual surface cues and, we hypothesize, the deeper fact that retrieval-augmented answers are more grounded and complete and are thus recognizable to experts. Because blinding failed, we do not rely on the human ratings for causal inference; we instead use rater-independent measures (Section 2.6.5 and Section 3.5.3) and report the human ratings as corroboration. Re-scoring used a separate regime (for example, Custom GPT re-scored 4.78 vs. 3.80 originally, an upward shift consistent with the identified expectation bias); we therefore analyze the paired experiment only within-round. High inter-rater reliability (ICC 0.965) reflects within-round consistency among raters and does not address the blinding failure or the between-round drift. For clarity of counts, the original study comprised 900 responses (6 systems × 50 questions × 3 replicates); the paired round re-scored 650 arm-question responses (13 arms × 50); and the automated judge (Section 2.6.5) scored 1100 responses. The 13 arms comprise the five base models, their five guideline-retrieval (+RAG) counterparts, and the Custom GPT, ConsensusGPT, and oracle-retrieval conditions.

#### 2.6.5. Automated Evaluation

To obtain an accuracy and safety measure that is not produced by the (unblinded) human raters, each response was additionally scored by an automated judge—Claude Sonnet 5 (Anthropic; model identifier claude-sonnet-5), a large language model from a different developer than any evaluated system and therefore not itself under evaluation—accessed via API at temperature 0 on 12 July 2026. The judge was presented only with the question, the gold-standard answer, and the response, blind to the model and condition, rated accuracy on the same 1–5 rubric, and flagged content-level hallucination, defined as a claim that contradicts the gold or established knowledge or fabricates specifics, explicitly independent of whether a citation was present. Judge scores were validated against the three experts on the 650 core responses: Spearman ρ = 0.81, 95.7% within-one agreement, with the judge slightly more conservative than the experts (mean 4.24 vs. 4.41) and concordant hallucination rates (18.2% vs. 16.5%). Two features of this validation require explicit statement. First, the judge scored the same citation-normalized text that was presented to the human raters, so the source-identifying citations that constituted the principal surface tell were absent from its input as well. Second, and more fundamentally, agreement with the human experts cannot establish that the judge is free of their bias: the raters against whom it was calibrated are the same raters subsequently shown to identify the retrieval condition at 98.5%, so a high correlation is equally consistent with the judge sharing their expectation bias, and automated judges are independently known to favor verbosity, elaborate structure, and apparent confidence when scoring text. The observed agreement therefore establishes that the judge is calibrated to the same scale as the experts, not that it is an independent arbiter. Accordingly, we rank the evidence by its independence from human and model judgment: the deterministic, log-derived retrieval metrics (citation groundedness and recall@5) are the primary rater-independent evidence; the judge’s binary hallucination flag, anchored to the gold-standard answer and defined independently of citation presence, is the principal safety measure; and the judge’s graded 1–5 accuracy scores, together with the human ratings, are reported as supporting evidence. Throughout, we use rater-independent to mean that a measure is not produced by the human raters; as the preceding paragraph makes clear, it does not imply freedom from the biases those raters exhibited, which is why the deterministic metrics rather than the judge carry the causal weight. The exact judge prompt is released with the code.

### 2.7. Statistical Analysis

#### 2.7.1. Sample Size Calculation

Sample size was determined a priori to detect clinically meaningful differences in accuracy scores between LLMs. For the primary Kruskal–Wallis analysis with six groups, α = 0.05, and power = 0.80, assuming a medium-to-large effect size (Cohen’s f = 0.30), a minimum of 108 observations per group was required (G*Power 3.1). With 50 questions × 3 replicates = 150 observations per LLM (total N = 900), we exceeded this requirement by 39%. This calculation assumed independent observations; the confirmatory mixed-effects models (Section 2.7.6) verify that the clustered structure did not affect the primary conclusions, which the observed effect—far exceeding the powering assumption—supported.

#### 2.7.2. Inter-Rater Reliability

Inter-rater reliability was assessed using the intraclass correlation coefficient (ICC) for average measures, specifically ICC(2,k) based on a two-way random-effects model. This approach accounts for both systematic differences between raters and random measurement error. Pre-specified thresholds for acceptable reliability were: ICC < 0.50 (poor), 0.50–0.75 (moderate), 0.75–0.90 (good), >0.90 (excellent). Pairwise Spearman rank correlations between reviewers were calculated as supplementary reliability indicators.

#### 2.7.3. Cross-Model Comparison

Normality of score distributions was assessed using the Shapiro–Wilk test. As Likert-scale data violated normality assumptions (all *p* < 0.001), non-parametric methods were employed. This cross-model comparison used the Kruskal–Wallis H-test to compare accuracy, comprehensiveness, and clarity scores across the six LLMs. Effect size was quantified using eta-squared (η^2^), calculated as η^2^ = (H − k + 1)/(N − k), where H is the Kruskal–Wallis statistic, k is the number of groups, and N is the total sample size. Effect size interpretation followed Cohen’s conventions: η^2^ < 0.06 (small), 0.06–0.14 (medium), >0.14 (large). Robustness of all primary comparisons was subsequently confirmed using mixed-effects models that account for question-level and question-by-model clustering (Section 2.7.6).

#### 2.7.4. Post Hoc Pairwise Comparisons

Where the omnibus Kruskal–Wallis test indicated significant between-group differences, post hoc pairwise comparisons were performed using Dunn’s test with Bonferroni correction for multiple comparisons. With 6 groups, 15 pairwise comparisons were conducted, yielding an adjusted significance threshold of α = 0.05/15 = 0.0033.

#### 2.7.5. Pre-Specified Secondary Analyses

The following secondary analyses were pre-specified in the study protocol:

1. RAG vs. Non-RAG Comparison: Mann–Whitney U test comparing Custom GPT (RAG) against all other models combined, with rank-biserial correlation (r) as effect size measure.
2. Difficulty Subgroup Analysis: Stratified Kruskal–Wallis tests within each difficulty level (easy, medium, hard) to assess whether LLM performance differences vary by question complexity.
3. Question Type Subgroup Analysis: Separate Kruskal–Wallis tests for each question type (factual, recommendation-level, clinical decision, case vignette).
4. Hallucination Rate Comparison: Chi-square test comparing hallucination frequencies across LLMs.

#### 2.7.6. Mixed-Effects Sensitivity Analysis

Because the response-level observations were clustered—each of the 50 questions was answered by all six models, with three replicates per model—we performed a confirmatory mixed-effects analysis. For each response, the three reviewer ratings were averaged (yielding 900 response-level scores from 2700 individual ratings), and linear mixed-effects models of the form score ~ LLM + (1|question) + (1|question:LLM) were fitted. Random intercepts for question captured systematic differences in question difficulty, and random intercepts for the question-by-model combination absorbed the replicate-level dependency. Models were fitted by restricted maximum likelihood (REML) for estimation of variance components and marginal means; for likelihood-ratio tests of the fixed model effect, nested models were refitted by maximum likelihood. Estimated marginal means and the pre-specified contrast of Custom GPT (RAG) versus the mean of the five comparator models were computed with Kenward–Roger-adjusted degrees of freedom. As an ordinal sensitivity analysis, a cumulative link mixed model was fitted to the 2700 individual reviewer ratings with crossed random intercepts for question and reviewer.

#### 2.7.7. Software and Reproducibility

All statistical analyses were performed using Python 3.11 with the following packages: pandas 2.0, scipy 1.11, pingouin 0.5.3 (ICC calculation), scikit-posthocs 0.8.0 (Dunn’s test), matplotlib 3.8, and seaborn 0.13 (visualization). Mixed-effects models were fitted in R 4.5.3 using the lme4 (1.1.37), emmeans (1.11.2; Kenward–Roger approximation via pbkrtest 0.5.5), and ordinal (2023.12-4.1) packages. Statistical significance was defined as *p* < 0.05 (two-tailed) unless otherwise specified.

#### 2.7.8. Paired and Automated Analysis

Our primary analysis isolates the effect of guideline retrieval within each base model. Judge and human scores were each analyzed with a linear mixed model (score ~ condition × model + (1|question), with an additional (1|question:model) term for the three-replicate human data) using Kenward-Roger degrees of freedom; the within-model contrast (RAG − base) per model estimates the retrieval effect with base capability held constant, and the condition × model interaction quantifies heterogeneity across bases. Retrieval quality was summarized by recall@5 against gold references, and an oracle-retrieval condition (gold section supplied directly, one model) provided an existence-proof ceiling. Citation groundedness and faithfulness were computed deterministically from the logs. To test directly whether an automated judge simply rewards longer answers, we computed the length of every scored response and refitted the paired models with length as a covariate, and examined the length-accuracy association within and across conditions. Because scores are bounded and near-ceiling for strong bases, we flag ceiling compression as an alternative reading of the condition × model interaction.

## 3. Results

### 3.1. Study Population and Data Collection

A total of 900 query-response pairs were generated and evaluated, each independently rated by three reviewers (2700 individual ratings in total), comprising 50 clinical questions derived from the German S3 guideline for oral cavity carcinoma, each presented to six different LLMs with three replicates per model (50 × 6 × 3 = 900). The questions covered 17 distinct guideline domains, including risk factors, screening, diagnostics, staging, surgical therapy, radiotherapy, and follow-up care. Question types included factual questions (n = 396, 44.0%), recommendation-level questions (n = 306, 34.0%), clinical decision questions (n = 108, 12.0%), and case vignettes (n = 90, 10.0%). Difficulty levels were distributed as easy (n = 162, 18.0%), medium (n = 414, 46.0%), and hard (n = 324, 36.0%). The complete response-level dataset and the underlying question catalogue are provided in the Supplementary Materials (Tables S1 and S2).

### 3.2. Inter-Rater Reliability

Three independent reviewers evaluated all LLM responses using a standardized 5-point Likert scale (Table 4). Inter-rater reliability was assessed using the intraclass correlation coefficient (ICC) for average measures. Excellent agreement was observed for accuracy (ICC = 0.959, 95% CI [0.89, 0.98]) and comprehensiveness (ICC = 0.968, 95% CI [0.91, 0.98]). Good agreement was found for clarity (ICC = 0.737, 95% CI [0.67, 0.79]). Pairwise Spearman correlations between reviewers ranged from ρ = 0.902 to ρ = 0.927 for accuracy ratings, indicating high consistency in evaluation. These results exceed the pre-specified threshold for excellent reliability (ICC > 0.90), indicating excellent within-round agreement among the raters.

### 3.3. Cross-Model Comparison (Original, Descriptive Round)

Significant differences in performance were observed across the six LLMs (Table 5). Custom GPT with retrieval-augmented generation (RAG) achieved the highest accuracy score (3.80 ± 1.22), followed by ConsensusGPT (3.50 ± 1.20). The remaining models without guideline access demonstrated substantially lower accuracy: DeepSeek-V3.2-Exp (2.86 ± 1.18), Mistral Small 3.2 (2.63 ± 1.05), Qwen3-Next-80B (2.62 ± 1.06), and GPT-OSS-120B (2.54 ± 1.09) (Table 5).

The Kruskal–Wallis H-test revealed statistically significant differences among LLMs for all outcome measures: accuracy (H(5) = 218.08, *p* < 0.001, η^2^ = 0.238), comprehensiveness (H(5) = 51.87, *p* < 0.001, η^2^ = 0.052), and clarity (H(5) = 40.58, *p* < 0.001, η^2^ = 0.040). The effect size for accuracy was large (η^2^ = 0.238), indicating that model selection explains approximately 24% of the variance in accuracy scores.

Because a pre-specified manipulation check found that raters identified retrieval-augmented answers with 98.5% accuracy, this cross-model comparison was not effectively blinded and is reported as descriptive context; causal inference rests on the self-controlled within-model experiment (Section 3.3.4) and the rater-independent measures (Section 3.5.3). The Hallucination and Sources columns derive from the same human-rater round; the content-level hallucination metric in Section 3.5.3 (a reduction from 42% to 4% with guideline retrieval) supersedes the hallucination values shown here.

#### 3.3.1. Post Hoc Pairwise Comparisons

Dunn’s post hoc test with Bonferroni correction revealed that Custom GPT (RAG) and ConsensusGPT significantly outperformed all other models in accuracy (*p* < 0.001). No significant difference was observed between Custom GPT and ConsensusGPT (*p* = 0.471). Similarly, no significant differences were found among the four models without guideline access (DeepSeek, Mistral, Qwen3, GPT-OSS; all pairwise *p* > 0.05), suggesting comparable baseline performance when LLMs lack domain-specific knowledge retrieval.

#### 3.3.2. RAG-Enhanced vs. Standard LLMs

A pre-specified analysis comparing Custom GPT (RAG) against all other models combined showed higher scores across all metrics in this original, unblinded comparison. For accuracy, Custom GPT achieved a mean score of 3.80 compared to 2.83 for non-RAG models (Mann–Whitney U = 87,034, *p* < 0.001, effect size r = 0.547). In this original round the guideline-augmented model scored 34% higher; because this cross-model comparison was not effectively blinded (Section 2.6.4), we report it as descriptive context and base causal inference on the within-model analysis (Section 3.3.4; Table 6, pooled +0.64).

#### 3.3.3. Robustness to the Clustered Data Structure

To verify that the observed differences were not an artifact of treating repeated evaluations as independent, we refitted the data with linear mixed-effects models incorporating crossed random effects for question and question-by-model. Question-level clustering was substantial—random intercepts for question accounted for 70.9% of the total variance in accuracy (ICC = 0.71)—confirming the need to model this dependency. After accounting for clustering, the effect of model remained highly significant for accuracy (χ^2^(5) = 161.9, *p* < 0.001), comprehensiveness (χ^2^(5) = 47.8, *p* < 0.001), and clarity (χ^2^(5) = 26.5, *p* < 0.001), and the model-estimated marginal means matched the descriptive values in Table 5 to two decimal places. The pre-specified contrast confirmed that Custom GPT (RAG) significantly outperformed the mean of the five comparator models for accuracy (adjusted mean difference = 0.97 points, 95% CI 0.80–1.14, *p* < 0.001), comprehensiveness (0.36, 95% CI 0.18–0.55, *p* < 0.001), and clarity (0.08, 95% CI 0.02–0.15, *p* = 0.009). Consistent with the post hoc analysis (Section 3.3.1), the two guideline- or literature-augmented models (Custom GPT and ConsensusGPT) again ranked highest, and an ordinal cumulative link mixed model fitted to the individual reviewer ratings yielded the same overall conclusion, confirming a significant advantage of Custom GPT (RAG) over the comparator group. The cross-model comparison was therefore robust to the modeling of within-question and within-reviewer dependence; because that comparison was not effectively blinded (Section 2.6.4), it is reported as descriptive context, and causal inference rests on the within-model analysis (Section 3.3.4).

#### 3.3.4. Isolating the RAG Effect: Within-Model Comparison

**Figure 1:**
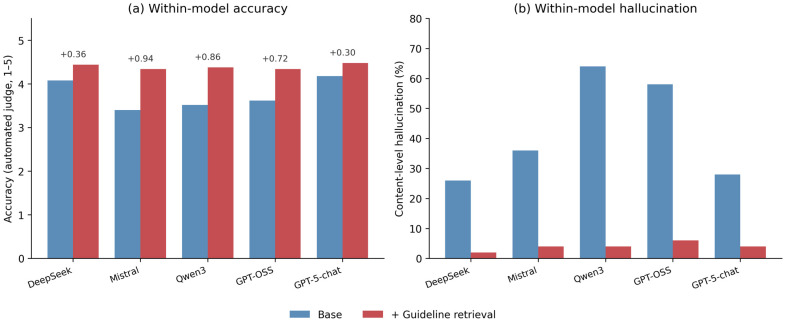
Within-model effect of guideline retrieval (automated judge): (**a**) accuracy and (**b**) content-level hallucination, base vs. + guideline retrieval, for each base model.

On the automated judge, holding each base model fixed, adding guideline retrieval improved accuracy by a pooled +0.64 points (Figure 1; 95% CI 0.47–0.80, *p* < 0.001; Table 6). The effect differed across bases (condition × model interaction χ^2^(4) = 9.9, *p* = 0.04): it was large for the weaker models—Mistral +0.94 (0.58–1.30), Qwen3 +0.86 (0.50–1.22), and GPT-OSS +0.72 (0.36–1.08)—and small and not statistically significant for the already-strong bases, DeepSeek +0.36 (−0.01–0.72) and GPT-5 +0.30 (−0.07–0.66), a pattern partly attributable to ceiling compression that we do not interpret mechanistically. The human ratings reproduced the pooled effect (+0.61, 0.49–0.74). GPT-5 run through the transparent pipeline showed no significant difference from the proprietary Custom GPT (judge accuracy 4.48 vs. 4.58; difference −0.10, 95% CI −0.41–0.21; human 4.71 vs. 4.78); no equivalence margin was pre-specified, and the interval does not exclude a modest difference in either direction, showing the flagship result is reproducible without the vendor interface.

On 25 additional primary-guideline questions, selected from the guideline’s recommendations and independently verified against the source by two oral-and-maxillofacial surgeons (100% agreement), the judge found a pooled retrieval effect of +1.11 points (95% CI 0.92–1.30, *p* < 0.001), with hallucination falling from 32% to 1%, confirming that the effect generalizes to new items (Table 7).

### 3.4. Subgroup Analyses

#### 3.4.1. Performance by Question Difficulty

**Figure 2:**
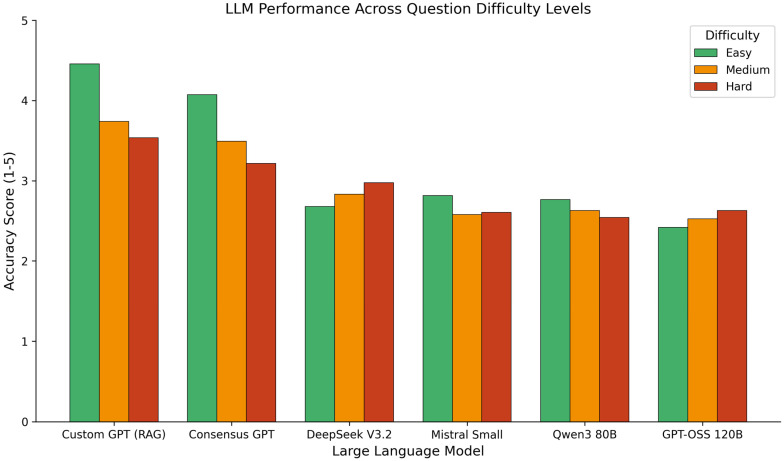
Performance by difficulty level (Easy/Medium/Hard).

LLM performance varied substantially across difficulty levels (Figure 2) (Table 8). For easy questions, Custom GPT achieved near-optimal accuracy (4.46 ± 0.93), while non-RAG models scored considerably lower (range: 2.42–2.81). Notably, the performance gap between RAG and non-RAG models narrowed for hard questions: Custom GPT scored 3.54 ± 1.24 compared to 2.54–2.98 for other models. This suggests that while RAG provides consistent advantages, complex clinical scenarios remain challenging even with guideline access.

#### 3.4.2. Performance by Question Type and Domains

Question-type comparisons below are based on adequate cell sizes, whereas the domain-level patterns involve only two to four items per domain and are therefore reported as exploratory, hypothesis-generating observations rather than as a basis for statistical inference.

**Figure 3:**
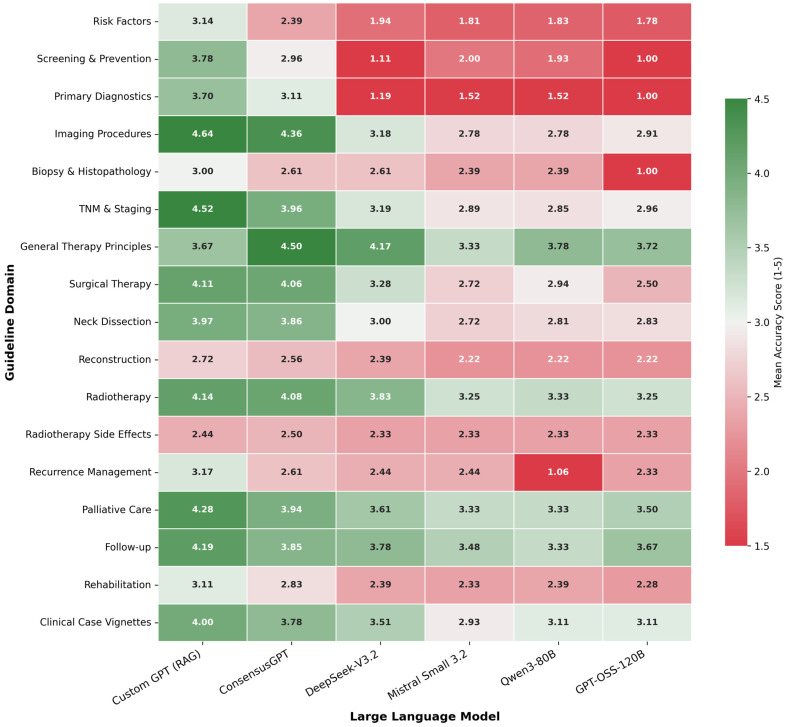
Exploratory heatmap of mean domain-level accuracy across the 17 guideline domains (2–4 items per domain). Because of the small number of items per domain, these patterns are shown for hypothesis generation only and are not used for inference.

Significant between-group differences were observed across all question types (all *p* < 0.001 by Kruskal–Wallis test). The largest effect was seen for recommendation-level questions (H = 123.78, *p* < 0.001), where accurate recall of specific guideline recommendations and evidence grades was critical. Case vignette questions, requiring integration of multiple guideline aspects, also showed substantial model differentiation (H = 55.32, *p* < 0.001). Performance varied considerably across the 17 guideline domains (Figure 3). Custom GPT (RAG) demonstrated consistently high accuracy across most domains, with particularly strong performance in Imaging Procedures (4.64), TNM and Staging (4.52), and Palliative Care (4.28). In contrast, all models struggled with certain specialized domains, including Reconstruction (range 2.22–2.72), Radiotherapy Side Effects (2.33–2.50), and Risk Factors (1.78–3.14), suggesting these areas may require more nuanced or specialized knowledge not adequately captured by current LLMs.

Notably, the RAG advantage was most pronounced in domains with clearly codified guideline recommendations, such as Surgical Therapy (Custom GPT: 4.11 vs. non-RAG mean: 2.90) and Follow-up protocols (Custom GPT: 4.19 vs. non-RAG mean: 3.42). Domains requiring synthesis of scattered information or interpretation of complex clinical scenarios, such as Recurrence Management, showed reduced performance across all models.

### 3.5. Safety and Quality Metrics

#### 3.5.1. Hallucination Detection (Human-Rater Round)

**Figure 4:**
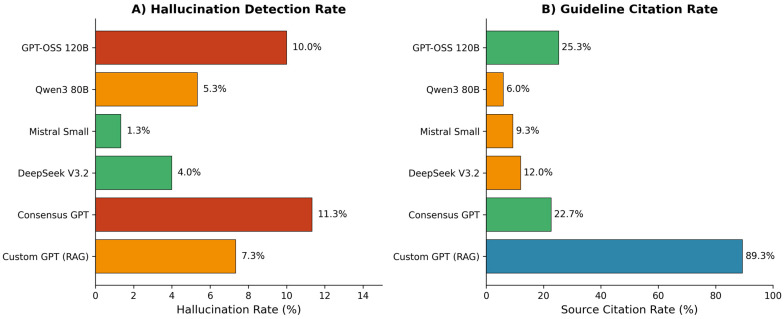
Hallucination and source citation rates from the human-rater round (Section 2.6.4); the content-level hallucination metric is shown in Figure 1b and Section 3.5.3.

Hallucination rates varied significantly across models (χ^2^(5) = 17.29, *p* = 0.004; Figure 4). Mistral Small 3.2 demonstrated the lowest hallucination rate (1.3%), followed by DeepSeek-V3.2-Exp (4.0%) and Qwen3-Next-80B (5.3%). Notably, Custom GPT with RAG showed a moderate hallucination rate (7.3%), potentially attributable to occasional misinterpretation of retrieved guideline content. ConsensusGPT (11.3%) and GPT-OSS-120B (10.0%) exhibited the highest hallucination frequencies, raising concerns about reliability in clinical decision support contexts.

#### 3.5.2. Guideline Citation Behavior

A striking difference emerged in source citation patterns. Custom GPT (RAG) cited the S3 guideline in 89.3% of responses, providing specific chapter references, recommendation numbers, and evidence levels. In contrast, non-RAG models rarely cited authoritative sources, with rates ranging from 6.0% (Qwen3) to 25.3% (GPT-OSS-120B). This disparity highlights the transparency advantage of RAG systems, enabling clinicians to verify AI-generated recommendations against primary sources.

#### 3.5.3. Results Not Produced by the Human Raters

We order these measures by their independence from human and model judgment. The deterministic metrics are computed directly from the retrieval logs and involve no rater or model judgement at all: citation groundedness rose within models from 0% to 51–89%, and retrieval recall@5 against gold references was 92%. We interpret groundedness as evidence of auditability and faithfulness rather than as independent proof of an accuracy gain, because a base model without retrieved context cannot, by construction, cite a location present in that context. Next, content-level hallucination—flagged by the judge against the gold-standard answer, and therefore model-assigned rather than deterministic, but binary and explicitly independent of whether a citation was present—fell within models from a pooled 42% to 4% (DeepSeek 26→2, Mistral 36→4, Qwen3 64→4, GPT-OSS 58→6, GPT-5 28→4), a large and consistent safety benefit across all bases, including those already near the accuracy ceiling. Finally, the graded 1–5 judge accuracy scores show the within-model retrieval gains reported in Section 3.3.4; being graded rather than binary, they are the measure most exposed to the known stylistic preferences of automated judges and are reported as supporting rather than primary evidence. The oracle-retrieval condition reached 4.96 accuracy with 0% hallucination for DeepSeek (a single-model existence proof, not a general ceiling). Item-level attribution indicated that a material share of the residual base-to-oracle headroom was recovered by supplying the correct context, so residual error traces to both retrieval and generation rather than predominantly to generation. Response length does not explain away the judge’s accuracy gain, and the association runs opposite to the direction a verbosity preference would predict. Guideline retrieval made answers substantially shorter rather than longer (mean 484 words at base vs. 264 with retrieval; the retrieval-augmented answer was the longer of the two in only 4.0% of the 250 question-by-model pairs), and greater length was associated with lower, not higher, judge accuracy (Spearman ρ = −0.23, *p* < 0.001 pooled across conditions; ρ = −0.18, *p* = 0.003 within the retrieval arm and ρ = +0.04, *p* = 0.52 within the base arm). Refitting the paired model with length as a covariate attenuated but did not remove the retrieval effect (pooled +0.48 points, 95% CI 0.22–0.73, *p* < 0.001, vs. +0.64 unadjusted); this adjustment is conservative, because length is itself changed by retrieval and is thus a consequence of the intervention rather than a confounder. The hallucination reduction was likewise robust to length adjustment (odds ratio 0.06, 95% CI 0.03–0.14, *p* < 0.001), with longer responses independently more likely to contain a hallucination.

### 3.6. Second-Guideline Pilot (Exploratory)

On an independent AWMF S3 guideline within the same specialty family (antiresorptive-associated jaw necrosis, MRONJ; 007-091), 20 expert-selected and independently verified questions (100% two-reviewer agreement) were processed through the identical pipeline. On the automated judge, retrieval improved accuracy by a pooled +1.07 points (95% CI 0.85–1.29, *p* < 0.001) and reduced content-level hallucination from 40% to 4%, directionally consistent with, and somewhat larger than, the primary guideline. Given its size and same-specialty scope, we interpret it as preliminary robustness evidence to a second guideline, not as multi-specialty or multi-language generalization, which remain future work.

## 4. Discussion

This prospective benchmark study provides one of the first systematic comparisons of retrieval-augmented generation (RAG) versus standard large language models for answering clinical questions derived from a German S3 guideline in oral and maxillofacial surgery. Holding each base model fixed and using an automated judge, our findings show that adding guideline retrieval improved accuracy by a pooled +0.64 points (95% CI 0.47–0.80)—concentrated in the weaker base models and small or non-significant for the already-strong ones—and reduced content-level hallucination from 42% to 4%. The original cross-model comparison, in which the guideline-augmented model scored 34% higher, motivated this follow-up but is reported as descriptive context because a pre-specified check found that retrieval-augmented answers remained identifiable to expert raters (Section 2.6.4). The large effect size (η^2^ = 0.238) reflects the spread across models in this descriptive, unblinded cross-model comparison; because that comparison was not effectively blinded, the clinically relevant conclusion—that guideline retrieval improves accuracy and safety—rests on the within-model analysis and, within it, primarily on the deterministic retrieval metrics (Section 3.3.4 and Section 3.5.3). These results align with recent meta-analyses demonstrating that RAG significantly improves LLM performance in biomedical applications, with pooled odds ratios of 1.35 (95% CI 1.19–1.53) favoring RAG-enhanced systems [14].

Our findings are consistent with the broader literature on LLM performance in medical examinations and clinical reasoning tasks. Studies evaluating GPT-4 on the United States Medical Licensing Examination (USMLE) have reported accuracy rates of 85–90% [22,23], substantially higher than the 2.54–2.86 mean scores on the original human scale (the same models scored 3.40–4.08 on the automated judge, Table 6) observed for non-RAG models in our study. This discrepancy likely reflects the specialized nature of our question set, which required precise recall of guideline-specific recommendations, evidence grades, and domain-specific terminology that standard LLMs may not have encountered during pre-training. In contrast, the Custom GPT with direct guideline access achieved accuracy scores comparable to those reported for RAG-enhanced systems in hepatology guideline interpretation, where accuracies exceeding 96% were observed [15]. The performance advantage of RAG systems was most pronounced for recommendation-level questions requiring identification of specific evidence grades and recommendation strengths, consistent with prior observations that RAG particularly benefits tasks requiring precise factual retrieval from authoritative sources [13].

On the content-level hallucination metric (Section 3.5.3), adding guideline retrieval reduced hallucination within every base model, from a pooled 42% to 4%—an approximately ten-fold reduction that is the study’s principal safety finding. The original round’s narrower reviewer-flagged rates (1.3–11.3%, including 7.3% for the guideline-augmented Custom GPT) are reported as descriptive context and are not directly comparable. Retrieval thus reduces, but does not eliminate, hallucination. This finding aligns with systematic reviews documenting hallucination rates ranging from 1.5% in optimized clinical workflows to over 28% for citation generation tasks [17,18]. The persistence of hallucinations even in RAG systems may reflect misinterpretation of retrieved content, incomplete retrieval of relevant guideline sections, or generation of plausible-sounding but incorrect inferences from partial information [21]. These observations reinforce that human oversight remains essential when deploying LLMs for clinical decision support, regardless of architectural enhancements. Recent studies have emphasized that current state-of-the-art LLMs do not reliably adhere to diagnostic or treatment guidelines without external grounding, highlighting the continued need for verification mechanisms [24].

A striking finding was the dramatic difference in source citation patterns between RAG and non-RAG models. Custom GPT cited the S3 guideline in 89.3% of responses, providing specific chapter references and recommendation numbers, whereas non-RAG models rarely cited authoritative sources (6.0–25.3%). This transparency advantage represents a critical consideration for clinical implementation, as citation of primary sources enables clinicians to verify AI-generated recommendations against authoritative guidelines [12]. The ability to trace recommendations back to specific guideline sections addresses a key concern regarding LLM deployment in healthcare: the lack of explainability and transparency in AI-generated clinical advice [3]. Furthermore, structured citations facilitate appropriate trust calibration, allowing physicians to weigh AI recommendations based on the quality of supporting evidence rather than accepting outputs uncritically [25].

Our findings have several implications for the deployment of LLMs in clinical decision support for oral and maxillofacial surgery. First, the substantial performance advantage of RAG-enhanced systems suggests that healthcare institutions seeking to implement LLM-based guideline assistants should prioritize architectures that incorporate authoritative knowledge bases rather than relying on pre-trained knowledge alone. Second, the persistence of hallucinations across all models necessitates implementation of human-in-the-loop verification, where clinicians review and validate AI-generated recommendations before clinical application [26]. Third, the observation that performance gaps narrowed for complex clinical scenarios indicates that LLMs—even RAG-enhanced systems—should be viewed as decision support tools rather than autonomous decision-makers, particularly for challenging cases requiring synthesis of multiple guideline aspects [27]. The collaborative model where AI provides initial recommendations with source citations, subject to physician review and modification, appears most aligned with current evidence on effective human-AI collaboration in healthcare [28].

Several limitations warrant consideration. First, our study focused on a single German S3 guideline, and generalizability to other guidelines, languages, or medical specialties requires further investigation. Second, the evaluation was conducted in a controlled research setting rather than integrated clinical workflows, where factors such as time pressure, incomplete patient information, and competing clinical demands may influence LLM utility. Third, inter-rater reliability was high (ICC > 0.95), but this reflects within-round consistency among raters rather than the validity of the comparison itself. Fourth, our expert reviewers were all oral and maxillofacial surgeons, potentially limiting assessment of interdisciplinary perspectives. Fifth, the rapidly evolving landscape of LLM development means that newer models may demonstrate different performance characteristics than those evaluated here. Sixth, the use of a small expert-reviewer panel in a controlled research setting, while providing consistent evaluation standards, may not fully capture the full variability of real-world, multidisciplinary clinical judgment.

Future research should investigate several key areas. Comparative evaluations across multiple guidelines and medical specialties would establish the generalizability of RAG advantages observed in our study. Prospective implementation studies integrating LLM decision support into clinical workflows could assess real-world utility, physician acceptance, and patient outcomes. Development of standardized evaluation frameworks and benchmarks specific to guideline-based medical question answering would facilitate reproducible comparisons across studies [29]. Additionally, research into hybrid approaches combining RAG with fine-tuning on domain-specific corpora may further enhance performance while mitigating hallucination risks [30]. As healthcare organizations increasingly consider LLM deployment, rigorous evaluation against authoritative clinical guidelines provides an essential foundation for responsible implementation [2].

Our contribution is not the unsurprising observation that a model supplied with the target guideline scores higher on that guideline. By holding each base model fixed we partition the advantage into a base-independent retrieval effect and residual base capability, and we show that the accuracy benefit is base-dependent—large for weaker models and modest for already-strong ones near the ceiling—whereas the reductions in hallucination and the gains in auditable, resolvable citation were consistent across all bases. Because blinded human evaluation could not be achieved, the weight of these conclusions rests on the deterministic retrieval metrics and on the reduction in content-level hallucination, with the automated judge’s graded accuracy scores reported as supporting evidence. Any accuracy benefit of external retrieval is a present-deployment advantage that may diminish as base models improve or internalize such content; the durable contribution is auditability—traceability of each recommendation to a versioned, citable guideline—which does not depend on base-model progress.

Several limitations warrant emphasis. Blinding was not achieved (98.5% identification) and human ratings drifted upward between rounds, so causal inference rests on the log-derived metrics and, secondarily, on the automated judge; the judge’s calibration is itself circular in one important respect: it was benchmarked against the same expert raters whose blinding had failed, so close agreement with them cannot demonstrate independence from their expectation bias, and automated judges are separately known to prefer verbose, confidently phrased, and heavily structured answers. We therefore do not rest the causal claim on the judge’s graded accuracy scores. Three considerations bound this concern without dispelling it: the judge scored the same citation-normalized text; the retrieval effect survived adjustment for response length, in a direction opposite to a verbosity preference (Section 3.5.3); and a uniform stylistic preference would be expected to raise scores similarly in every base, whereas the observed gain is concentrated in the weaker ones—though, as noted in Section 3.3.4, ceiling compression offers an alternative account of that same pattern, so we treat this third observation as suggestive rather than decisive. The deterministic groundedness and recall metrics are free of this concern altogether. The oracle-retrieval arm was single-model, evidence beyond the index guideline is limited to a single same-specialty pilot, the groundedness and recall metrics depend on a team-built gold question-to-section mapping (inter-annotator agreement reported), and hosted generation is bounded by API and model-version stability.

## 5. Conclusions

In conclusion, this self-controlled benchmark demonstrates that guideline retrieval yields a reproducible improvement in the safety and auditability of large language models on clinical guideline questions that was consistent across base models, together with accuracy gains concentrated in the weaker bases. Within each base model, grounded and resolvable citation rose from 0% to 51–89% and retrieval recall@5 reached 92%, both computed deterministically from the retrieval logs. On the automated judge, content-level hallucination fell from 42% to 4% across all bases. The automated judge’s graded accuracy scores, which we treat as supporting evidence because the judge was calibrated against raters whose blinding had failed, showed a pooled within-model gain of +0.64 points (95% CI 0.47–0.80), concentrated in the weaker base models and small or non-significant for the already-strong ones and robust to adjustment for response length; a transparent, openly released pipeline reproduced the proprietary result without the vendor interface. Because a pre-specified check found that retrieval-augmented answers remain identifiable to experts (blinding was not achieved), we base these conclusions on rater-independent measures and report the original cross-model human comparison as descriptive context. Residual hallucination continues to require physician oversight for safe deployment.

## Figures and Tables

**Table 1:** Distribution of Questions by Type and Difficulty.

Question Type	Easy	Medium	Hard	Total (%)
Factual	8	9	5	22 (44%)
Recommendation-level	1	10	6	17 (34%)
Clinical decision	0	3	3	6 (12%)
Case vignette	0	1	4	5 (10%)
Total	9 (18%)	23 (46%)	18 (36%)	50 (100%)

Note: Difficulty was assigned based on clinical complexity and the number of guideline sections required for a complete answer.

**Table 2:** Characteristics of Evaluated Large Language Models.

Model	Parameters	Access	Architecture	Knowledge Source
Custom GPT (RAG)	N/A	OpenAI	GPT-5 + RAG	S3 Guideline PDF
ConsensusGPT	N/A	ChatGPT Plugin	GPT-5 + Plugin	Scientific literature
DeepSeek-V3.2-Exp	685B (MoE)	DeepInfra API	Transformer MoE	Pre-training only
GPT-OSS-120B	120B	DeepInfra API	Transformer	Pre-training only
Qwen3-Next-80B	80B (3B active)	DeepInfra API	Transformer MoE	Pre-training only
Mistral Small 3.2	24B	DeepInfra API	Transformer	Pre-training only
GPT-5-chat	N/A	OpenAI API	GPT-5 (chat)	Pre-training (±S3 guideline; paired within-model arm)

Note: MoE = Mixture of Experts; RAG = Retrieval-Augmented Generation; N/A = Not publicly disclosed. Parameter counts reflect total model size; active parameters during inference may differ for MoE architectures.

**Table 3:** Evaluation Dimensions and Rating Criteria.

Dimension	Definition and Criteria
Accuracy	Factual correctness compared to gold standard. 1 = Completely incorrect or contradicts guideline; 2 = Mostly incorrect with some accurate elements; 3 = Partially correct, missing key information; 4 = Mostly correct with minor errors; 5 = Completely correct, aligns fully with guideline
Comprehensiveness	Completeness of coverage. 1 = Addresses <20% of relevant aspects; 2 = Addresses 20–40%; 3 = Addresses 40–60%; 4 = Addresses 60–80%; 5 = Addresses >80% of relevant aspects mentioned in gold standard
Clarity	Communication quality. 1 = Incomprehensible or severely disorganized; 2 = Difficult to follow, poor structure; 3 = Adequate clarity, some organizational issues; 4 = Clear and well-organized; 5 = Excellent clarity, logical flow, appropriate medical terminology

Note: Reviewers were provided with detailed anchor descriptions and example responses for each scale point during training.

**Table 4:** Inter-Rater Reliability Statistics.

Dimension	ICC(2,k)	95% CI	ρ (R1–R2)	ρ (R1–R3)	ρ (R2–R3)
Accuracy	0.959	[0.89, 0.98]	0.912	0.902	0.927
Comprehensiveness	0.968	[0.91, 0.98]	0.889	0.896	0.949
Clarity	0.737	[0.67, 0.79]	0.493	0.620	0.484

Note: ICC = Intraclass Correlation Coefficient; CI = Confidence Interval; ρ = Spearman correlation coefficient; R1–R3 = Reviewers 1–3.

**Table 5:** Performance Metrics by Large Language Model.

Model	n	Accuracy	Comprehen.	Clarity	Halluc. %	Sources %
Custom GPT (RAG)	150	3.80 ± 1.22	4.13 ± 1.33	4.89 ± 0.27	7.3	89.3
ConsensusGPT	150	3.50 ± 1.20	4.22 ± 1.34	4.92 ± 0.19	11.3	22.7
DeepSeek-V3.2-Exp	150	2.86 ± 1.18	3.72 ± 1.40	4.80 ± 0.34	4.0	12.0
Mistral Small 3.2	150	2.63 ± 1.05	3.68 ± 1.37	4.82 ± 0.31	1.3	9.3
Qwen3-Next-80B	150	2.62 ± 1.06	3.64 ± 1.42	4.76 ± 0.36	5.3	6.0
GPT-OSS-120B	150	2.54 ± 1.09	3.56 ± 1.44	4.76 ± 0.32	10.0	25.3

Note: Values are presented as mean ± SD. Comprehen. = Comprehensiveness; Halluc. = Hallucination rate; Sources = Guideline citation rate. RAG = Retrieval-Augmented Generation. Scores range from 1 (worst) to 5 (best).

**Table 6:** Within-model effect of guideline retrieval (automated judge). Accuracy on a 1–5 scale; Δ with 95% CI from a linear mixed model.

Model	Base	+RAG	Δ Accuracy (95% CI)	Groundedness (+RAG)	Hallucination (Base → +RAG)
DeepSeek-V3.2	4.08	4.44	+0.36 (−0.01–0.72)	65%	26% → 2%
Mistral Small 3.2	3.40	4.34	+0.94 (0.58–1.30)	52%	36% → 4%
Qwen3-Next-80B	3.52	4.38	+0.86 (0.50–1.22)	51%	64% → 4%
GPT-OSS-120B	3.62	4.34	+0.72 (0.36–1.08)	59%	58% → 6%
GPT-5-chat	4.18	4.48	+0.30 (−0.07–0.66)	89%	28% → 4%
Pooled	—	—	+0.64 (0.47–0.80)	—	42% → 4%

**Table 7:** Retrieval effect on 25 additional primary-guideline questions (automated judge).

Model	Base	+RAG	Δ Accuracy (95% CI)
DeepSeek-V3.2	3.68	4.92	+1.24 (0.81–1.67)
Mistral Small 3.2	3.28	4.52	+1.24 (0.81–1.67)
Qwen3-Next-80B	3.80	5.00	+1.20 (0.77–1.63)
GPT-OSS-120B	3.32	4.56	+1.24 (0.81–1.67)
GPT-5-chat	4.08	4.72	+0.64 (0.21–1.07)
Pooled	—	—	+1.11 (0.92–1.30)

**Table 8:** Accuracy Scores by Question Difficulty Level.

Model	Easy (n = 162)	Medium (n = 414)	Hard (n = 324)
Custom GPT (RAG)	4.46 ± 0.93	3.74 ± 1.23	3.54 ± 1.24
ConsensusGPT	4.07 ± 0.66	3.49 ± 1.30	3.22 ± 1.19
DeepSeek-V3.2-Exp	2.68 ± 1.25	2.83 ± 1.22	2.98 ± 1.11
Mistral Small 3.2	2.81 ± 0.96	2.58 ± 1.14	2.60 ± 0.99
Qwen3-Next-80B	2.77 ± 1.05	2.63 ± 1.12	2.54 ± 1.00
GPT-OSS-120B	2.42 ± 1.19	2.53 ± 1.16	2.63 ± 0.97

Note: Values are presented as mean ± SD. Scores range from 1 (worst) to 5 (best).

## Data Availability

The complete datasets—the original 900 human-rated responses, the 650 re-scored paired-round responses, the 1100 automated-judge scores, the 25 additional primary-guideline items, and the 20-question second-guideline (MRONJ) pilot—together with the full transparent retrieval pipeline (code, FAISS index, chunked guideline text, prompts, per-query retrieval logs, the citation normalizer with its token-level audit, and the automated-judge prompt and outputs), are openly available on Zenodo (DOI: 10.5281/zenodo.21342659; released under CC BY 4.0/MIT). Reviewer assessments and gold-standard answers are included in the deposit. The S3 guideline is publicly available from the AWMF (https://www.awmf.org/leitlinien/detail/ll/007-100OL.html) (accessed on 17 July 2026).

## Supplementary Materials

The following supporting information can be downloaded at: https://www.mdpi.com/article/10.3390/diagnostics16152456/s1, Table S1: Complete response-level dataset (workbook sheet "Hauptdaten")—all 900 LLM responses (six systems × 50 questions × 3 replicates) with the full response text, the corresponding question, gold-standard answer, guideline reference and evidence level, the independent accuracy, comprehensiveness and clarity ratings of each of the three expert reviewers, and the per-response hallucination and source-citation flags; Table S2: Question catalogue (workbook sheet "Fragenkatalog")—the 50 clinical questions with their guideline domain, question type, difficulty level, gold-standard answer, guideline reference and evidence level. Questions, model responses and domain labels are given in the original German.
